# Prevalence and Predictors of Self-Medication Practice Among Teachers’ Education Training College Students in Amhara Region, Ethiopia: A Cross-Sectional Study

**DOI:** 10.3389/fphar.2020.593764

**Published:** 2021-02-02

**Authors:** Abebe Basazn Mekuria, Eshetie Melese Birru, Melkamu Teshome Tesfa, Mestayet Geta, Zemene Demelesh Kifle, Tsegaw Amare

**Affiliations:** ^1^ Department of Pharmacology, School of Pharmacy, College of Medicine and Health Sciences, University of Gondar, Gondar, Ethiopia; ^2^ Department of Health Systems and Policy, Institute of Public Health, College of Medicine and Health Sciences, University of Gondar, Gondar, Ethiopia

**Keywords:** practice, self-medication, Gondar, student, Ethiopia

## Abstract

**Background:** Self-medication practice is the use of medications without healthcare professional requests. It can lead to inappropriate medication usage, wastage of resources, increased chance of microbial resistance, and adverse drug reactions. Therefore, this study aimed at assessing the prevalence and associated factors of self-medication practice among teachers’ education training college students in the Amhara region, Ethiopia.

**Methods:** A multicentre cross-sectional study was conducted on 344 teachers’ education training college students in the Amhara region, Ethiopia, from January 1 to February 28, 2020. Data on sociodemography, the practice of self-medication, and factors associated with self-medication practice were collected through a self-administered structured questionnaire. Systematic random sampling was used to select participants. Descriptive statistics and univariate and multivariate logistic regression analyses were done to determine various variables and factors associated with self-medication practice.

**Results:** Out of the 344 respondents, 234 (68.0%) practiced self-medication. The most commonly cited indication for self-medication practice was headache (75, 32.05%), followed by abdominal discomfort (53, 22.6%). The respondents who were older than 26 years of age (AOR: 2.47, 95% CI: 1.18–3.94), were in the third year of study (AOR: 3.14, 95% CI: 1.94–5.79), lived in urban residence (AOR: 2.97, 95% CI: 1.06–3.64), had accessibility to a nearby pharmacy (AOR: 2.12, 95% CI: 1.43–4.46), and had peer/family pressure (AOR: 2.34, 95% Cl: 1.53–3.56) were significantly associated with self-medication practice.

**Conclusion:** More than two-thirds of the study participants practiced self-medication. Being from an urban area, having access to a private pharmacy, and higher year of study positively affect self-medication practice.

## Introduction

According to the World Health Organization (WHO) definition, self-medication is the selection and use of medicines to treat self-recognized illnesses or symptoms (Organization, 2010). Inappropriate self-medication practice results in wastage of resources, increases the chance of drug resistance, and causes serious health problems such as adverse drug reactions, treatment failure, misuse of medications, and drug dependence (Bekele et al., 2016). Despite this, self-medication may reduce health costs and save the time spent waiting to see doctors for minor health problems (Leiet al. 2018; Karimy et al., 2019).

Currently, self-medication is becoming a worldwide public health problem. The study report showed that up to 80% of drugs in developing countries were purchased without a prescription (Gore and Madhavan 1994; Shokrzadeh et al., 2019). Similarly, the study conducted in Iran showed that more than two-thirds of individuals had a history of self-medication practice (Karimy et al., 2019; Shokrzadeh et al., 2019). Studies showed that most of the university and college students were practicing self-medication, for example, in Serbia (Lukovic et al., 2014), Nagara (Johnson et al., 2016), and South India (Xiao et al., 2011), 79.9%, 92.4%, and 78.6% of the students, respectively, practiced self-medication. Despite the increased practice of self-medication among students all over the world, the majority are unaware of the harmfulness of self-medication (Gore and Madhavan 1994; Johnson et al., 2016; Karimy et al., 2019).

Many studies reported the factors associated with self-medication practice such as young age (Garofalo et al., 2015), low level of education, previous experience of self-medication, lack of time to visit physicians, low income (Arrais et al., 2016; Helal and Abou-ElWafa 2017), urban residence, greater availability of the medical product, media exposure, the urgency of the problem, trivial health problems, unavailability of means of transport, ability to self-manage the symptoms, and increase of pharmaceuticals advertisements (Lei et al., 2018; Shokrzadeh et al., 2019). Accordingly, individuals practiced self-medication for different purposes. Studies reported that headache, fever, cough (Parakh et al., 2013), gastrointestinal diseases, respiratory tract infections, maternal/menstrual, eye diseases, skin diseases, injury, and sexually transmitted diseases were common indications of self-medication practice (Garofalo et al., 2015; Johnson et al., 2016; Kassie et al., 2018). In Ethiopia, some studies were conducted regarding self-medication practice among university students. However, no study was conducted among teachers’ education training college (TETC) students in Ethiopia (Ayalew and adherence 2017). Therefore, the current study aimed to explore the prevalence and associated factors of self-medication practice among TETC students in the Amhara region, Ethiopia.

## Methods and Materials

### Study Design and Area

A multicentre cross-sectional study was conducted in TETC of the Amhara region from January 1 to February 28, 2020. Amhara region is the second biggest administrative region in Ethiopia. This region has ten universities and seven TETCs. Currently, 12,206 students are taking their training in TETC in the Amhara region. This study was approved by the ethical committee of the School of Pharmacy, University of Gondar, with an approval number of SoP-318/2012. Informed verbal and written consent was obtained from study participants before data collection. The collected information from respondents was kept confidential.

### Sample Size Determination and Procedure

The source population of the study was all students at the Amhara region TETC, while the study population was those students who are studying at TETC during the data collection period. Regular undergraduate students who were available during the study period were included in the study, while students who were seriously ill and incapable of hearing and speaking during data collection were excluded. Single population proportion formula (n = [(Zα/2) 2 × P (1−P)]/(D/2)) was applied with the assumption of 95% confidence interval, 5% margin of error, the prevalence (p) of self-medication practice in Ethiopia (50%) (Ayalew and adherence, 2017), and 5% for possible nonresponse to determine a final adjusted sample size of 372. The number of students to be interviewed in each college was calculated based on proportion of the total number of students found in each college. Since the sample is homogeneous, a systematic random sampling method was used to recruit the final interviewed students. The interval was determined using the systematic sampling formula: interval (i) = total number of students in each college/total number of students to be interviewed in each college. Then, the first interviewed student (r) was selected randomly. The next interviewed student was selected by adding interval on the first sample (r) and we kept adding members in the sample (r, r + i, r+2i …) to recruit the interviewed students in each college.

### Data Collection Tools and Techniques

Data collection was performed by three final year pharmacy students through a self-administered questionnaire. The questionnaire was developed after a careful literature review of the published studies (Garofalo et al., 2015; Bekele et al., 2016; Johnson et al., 2016; Helal and Abou-ElWafa 2017; Kassie et al., 2018). First, it was prepared in English, translated into the local language (Amharic), and then back-translated to the English language to ensure consistency. The tool was further pretested on 18 students who were not included in the final analysis and relevant modifications were performed before the commencement of the actual data collection. Completeness and fulfillment of all questions were checked by the principal investigator and data collectors. The final questionnaire consisted of 17 items that were divided into three main sections. The first section was focused on the sociodemographic characteristics, including age, gender, department, years of study, monthly income, residence, and parents’ education level. The second section aimed to assess the prevalence of self-medication practice, reasons for self-medication practice, factors that promote self-medication, sources of supply, and information source for self-medication. The final section focused on the environmental characteristics of participants, including the distance of health institution, accessibility to pharmacy, presence of health professionals into the family, presence of medication at home, and peer/family pressure.

### Statistical Analysis

The final collected data were checked for completeness, and responses were entered into and analyzed using the Statistical Package for the Social Sciences software version 24.0 (SPSS v24.0) for Windows. Frequencies and percentages were used to express different variables; bivariant and multivariate logistic regression analyses were used to determine factors associated with self-medication practice. Univariate analysis was done and variables with a *p* value less than 0.2 were further taken to the multivariate logistic regression analysis for proper adjustment with the possible confounders. In this study, adjusted odds ratio (AOR) at 95% confidence interval (95% CI) with *p* value < 0.05 was considered statistically significant.

## Results

### Sociodemographic Characteristics

Out of the 372 respondents invited to participate, 344 responded to the questions giving a response rate of 92.5%. The mean age [standard deviation (SD)] of respondents was 20.96 (2.40) years, and half (175, 50.9%) of the respondents were female. Among the respondents, 127 (36.9%) were in the third year of study. More than half of the respondents (134, 56.4%) had 200–500 ETB monthly income. Regarding the study department, the most cited department was Art (85, 24.7%), followed by Natural Science (78, 22.7%). The majority of the participants (179, 52.1%) traveled more than 1 h to reach health institutions, whereas 193 (56.1%) had access to pharmacies. The detailed sociodemographic and self-medication related characteristics of the respondents are summarized in Table 1.

**TABLE 1:** Sociodemographic characteristics of respondents.

Variable	Frequency (%)	SM practice (n = 234)		AOR (95% CI)
		Yes (n)	No (n)	
Age group in years				
16–20	232 (67.4)	143	89	1
21–25	94 (27.3)	80	14	**2.47 (1.18**–3.94)*
≥26	18 (5.3)	11	7	**1.55 (1.19**–2.88)*
Sex				
Male	169 (49.1)	117	52	1
Female	175 (50.9)	117	58	1.11 (0.67–2.83)
Year of study		(n = 184)		
1^st^ year	98 (28.5)	40	58	1
2^nd^ year	119 (34.6)	66	53	1.20 (1.04–3.17)*
3^rd^ year	127 (36.9)	78	49	3.14 (1.94–5.79)*
Residence				
Rural	80 (23.3)	61	19	1
Urban	264 (76.7)	193	91	2.97 (1.06–3.64)*
Parents’ educational level				
Cannot read and write	138 (40.1)	94	44	1
NF education	87 (25.3)	61	26	1.16 (0.93–3.43)
Grades 1–8	85 (24.7)	56	27	1.41 (0.46–3.75)
Grades 9–12	18 (5.2)	11	7	1.47(1.46–3.21) *
≥diploma	16 (4.7)	10	8	**2.27(1.32**–3.71) *
Department				
Amharic language	16 (4.7)	9	7	1
English language	44 (12.8)	26	18	1.14 (0.65–2.42)
Mathematics	46 (13.4	20	26	1.97 (0.56–2.79)
Natural Science**	78 (22.7)	36	42	1.21 (0.47–2.24)
Art***	85 (24.7)	43	42	1.29 (0.34–2.75)
Social Science ****	75 (21.8)	52	23	1.85 (0.67–2.92)
Monthly income				
<200 ETB	111 (32.3)	62	49	1
200–500 ETB	134 (56.4)	74	60	1.22 (0.47–2.45)
>500 ETB	39 (11.3)	21	18	1.65 (0.66–3.45)
The distance of HI				
<30 min	39 (11.3)	18	21	1
30 min–1 h	126 (36.6)	72	54	1.12(1.01-2.43)*
>1 h	179 (52.1)	110	69	2.67(1.44-3.34)*
Access to pharmacy				
No	193 (56.1)	77	74	1
Yes	151 (43.9)	125	68	2.12(1.43-4.46)*
HP in their family				
Yes	72 (20.9)	27	45	1
No	272 (79.1)	187	85	1.45 (0.21-3.14)
Peer/family pressure				
Yes	212 (61.6)	134	78	2.34(1.53-3.56)*
No	132 (38.4)	60	72	1

SM: self-medication; NF: nonformal; HI: health institution; HP: health professional; ≥ diploma = parents’ education diploma or above diploma; *significant at *p* value < 0.05; Natural Science**: biology, physics, and chemistry; Art***: art generalist, music generalist, special needs, and physical education; Social Science ****: civics and ethics generalist, geography, amd history. ETB: Ethiopian birr (1 USD = 32 Ethiopian Birr).

### Prevalence, Source of Drug, and Indication of Self-Medication Practice

Of the total respondents, 234 (68.0%) practiced self-medication. The majority (184, 78.6%) of students started self-medication practice after they joined college. Among students who practice self-medication, 78 (33.3%) practiced more than four times and 72 (30.8%) faced adverse drug events. More than half of the students (135, 57.7%) did not read the instruction. From the total self-medication users, 171 (73.1%) got medication from the pharmacy and 89 (38.0%) from friends (Table 2).

**TABLE 2:** Prevalence and source of the drug for self-medication practice among TETC students, Amhara region, Ethiopia, 2020 (N = 344).

Statement	Answer	Frequency	%
Do you have ever practice self-medication in your lifetime?	Yes	234	68.0
	No	110	32.0
When you started to practice self-medication practices? (n = 234)	Before joining college	50	21.4
	After joining college	184	78.6
If your answer is yes for the above question, how many times practiced in your lifetime? (n = 234)	Once	33	14.1
	Twice	54	23.1
	Three times	69	29.5
	≥4 times	78	33.3
Duration of self-medication (n = 234)	For1 day	57	24.4
	For 2 days	52	22.2
	For 3 days	45	19.2
	Four days	33	14.1
	≥5 days	45	20.1
Do you always read the instruction? (n = 234)	Yes	99	42.3
	No	135	57.7
Do you have ever faced any adverse drug reaction? (n = 234)	Yes	72	30.8
	No	162	69.2
Where did you get that medication? (n = 234)*	Pharmacy	171	73.1
	Friends	89	38.0
	Family	72	30.8
	Others (specify	27	11.5

* Respondents have more than one choice.

**FIGURE 1:**
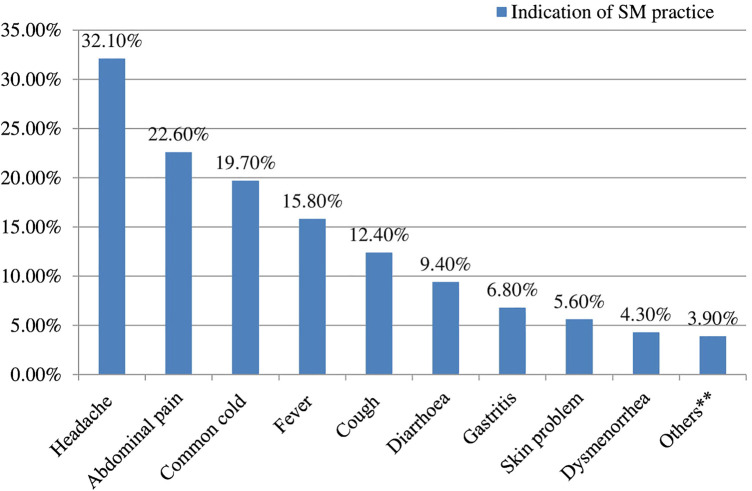
Indication of drugs for the self-mediation practice among students, Amhara region, Ethiopia, 2020 (n = 234)*.

In this study, the most commonly cited indication of self-medication was headache (75, 32.1%), followed by abdominal pain (53, 22.6%) and common cold (46, 19.7%). The indication of self-medication practice among respondents is summarized in Figure 1.

### Reasons for Self-Medication Practice

The most common reasons for choosing self-medication reported by the respondents were similarity of illness with previous illness (90, 38.9%), followed by nonserious disease (57, 24.4%) and ease of accessibility to drugs (40, 17.1%) (Table 3).

**TABLE 3:** Reasons for self-medication practice among TETC students, Amhara region, Ethiopia, 2020 (n = 234)*.

Variable	Frequency	Percent
The similarity of illness with previous illness	90	38.9
Disease not serious	57	24.4
Easily obtaining drugs	40	17.1
Friends’ suggestion	30	12.8
Self-medication being cheaper	29	12.4
Long waiting time in health service	23	9.8
Long distance to the health facility	22	9.4
Being embarrassed to talk about the disease	19	8.1
Affordability of the cost of drugs	14	6.0
Not trusting health professionals	10	4.3
Others	8	3.4

* Respondents have a chance to select more than one.

### Factors Associated With Self-Medication Practice Among Students

Variables that were significantly associated with self-medication practices in the bivariate analysis were further examined in multivariate logistic regression. The result showed that respondents aged 21–25 years were 2.47 times more likely to practice self-medication (AOR: 2.47, 95% CI: 1.18–3.94) than those aged16–20 years. Based on years of study, third-year students were 3.14 times more likely to practice self-medication (AOR: 3.14, 95% CI: 1.94–5.79) than first-year students. The odds of self-medication practice were 2.97 times higher among urban residents than among rural residents (AOR: 2.97, 95% CI: 1.06–3.64). Those who had accessibility to pharmacies were nearly two times (AOR: 2.12, 95% CI: 1.43–4.46) more likely to experience self-medication practice compared to those who had no accessibility to pharmacy. The odds of self-medication practice among respondents who had peer/family pressure was 2.34 times (AOR: 2.34, 95% Cl: 1.53–3.56) more likely to practice self-medication compared to those who had not peer pressure (Table 1).

## Discussion

Self-medication is a common practice in both developed and developing countries (Ayalew and adherence 2017). This may lead to incorrect selection of medication, harmful drug interactions, incorrect dosage, risk of drug dependence, and abuse (Mamo et al., 2018). Several studies revealed that self-medication practice is common practice throughout the world (Araia et al., 2019).The current study is designed to assess the prevalence and association factors of self-medication practice among TETC students in the Amhara region, Ethiopia. Furthermore, this study can provide baseline information for healthcare policymakers and education curriculum designers.

According to this study, 68.0% of students have practiced self-medication. This finding is nearly comparable with a study done at Addis Ababa, Ethiopia (75.5%) (Shafie et al., 2018), and Egypt (62.9%) (Helal and Abou-ElWafa 2017). However, it was lower than the findings from Nigeria (92.39%) (Johnson et al., 2016), Nepal (81.9%) (Badiger et al., 2012), Southwestern Nigeria (91.4%) (Osemene and Lamikanra 2012), and South India (84.6%) (Kumar et al., 2013). This might be due to the presence of advanced telemedicine services and awareness about medications. Another possible explanation may also due to the differences in sociodemographic factors and sample sizes. On the other hand, the finding is higher than those of the studies done in Jiangsu University (47.9%) (Zhu et al., 2016), West Bengal (43.24%) (Banerjee and Bhadury 2012), Mekelle University (43.24%) (Gutema et al., 2011), and Jimma University (45.9%) (Ararsa and Bekele 2015), Nekemte (36.7%) (Sado and Gedif 2014), Dire Dawa (41%) (Abrha et al., 2014), South India (35.9%) (Divya et al., 2016), Bahir Dar (23,3%) (Mihretie 2014), Sire town (27.16%) (Jaleta, Tesema et al., 2016), Siltʼe zone (24.40%) (Mossa et al., 2012), and Brazil (16%) (Arrais et al., 2016). The reason for the wide variation in the prevalence of self-medication practice might be due to the variation of social determinants of health, beliefs, and the culture of the population.

In this study, 30.8% were faced with adverse drug events. This was in line with a study done in North India (29.77%) (Parakh et al., 2013). However, the finding was higher than that in a study reported from South India (5.4%) (Badiger et al., 2012) and Eretria (9.2%) (Araia et al., 2019). Several previous studies revealed that pharmacy was the main source of medication and friends, relatives, and leftover medications from previous prescriptions were denoted as some of the frequently reported sources (Abay and Amelo 2010; Osemene and Lamikanra 2012; Ahmadi et al., 2016; Helal and Abou-ElWafa 2017). The present finding also showed similar results that pharmacy accounts for 73.1% as the main source for self-medication practice. Easy accessibility to drugs from pharmacies might be linked to the absence of clear legislation concerning access to medicine in Ethiopia. This legislation gap may attribute to the increment of the number of persons who might practice self-medication. Therefore, such practice may lead to irrational drug use and the development of drug resistance, possibly endangering human life.

The present study revealed that the commonest indication of self-medication practice was a headache (32.1%), followed by abdominal pain (22.6%). Some studies reported comparable findings in which headache was the leading indication of self-medication (Kumar et al., 2013; Shehnaz et al., 2014; Gyawali et al., 2015). Unlike the finding of this study, the common indications for self-medication were urinary tract infection (UTI), sore throat, and diarrhea in Nigeria (Osemene and Lamikanra 2012). A study conducted in Egypt revealed that cold, headache, sore throat, intestinal colic, and cramps were among the main diseases related to self-medication practice (Helal and Abou-ElWafa 2017), whereas a study conducted in Southwestern Nigeria reported that UTI, sore throat, and diarrhea were among the core diseases linked to self-medication practices (Osemene and Lamikanra 2012). This may be due to the difference in the prevalence of these diseases across the study area. In this study, the most commonly cited sources of drugs were pharmacy (73.1%), followed by family (38.2%). This finding is similar to the study done in India (Johnson et al., 2016).

In the current study, the topmost three reasons that led participants to practice self-medication were similarity of illness with previous illness (38.9%), feeling the disease is not serious (24.4%), and friends' suggestion (12.8%). This finding was similar to studies done in Jimma town (Ararsa and Bekele 2015), Iran (Jaleta et al., 2016), and Asella, Ethiopia (Bekele et al., 2016). In contrast, other studies reported previous experience as the first main reason for self-medication (Abay and Amelo 2010; Gutema et al., 2011; Abdi et al., 2018; Araia et al., 2019).

The multivariate analysis revealed that third-year students were 3.14 times more likely to practice self-medication (AOR: 3.14, CI: 1.94–5.79, *p* < 0.05) than first-year students. This finding is similar to the study done in Southwest Nigeria, Egypt, and Ethiopia that stated a significant association between years of study and self-medication (Osemene and Lamikanra 2012; Karthik et al., 2014; Helal and Abou-ElWafa 2017). However, a previous study conducted in Peru and Ethiopia did not show a significant association between the years of the study (Núñez et al., 2016; Gelayee, 2017). This may be associated with students’ awareness and knowledge about drugs and disease. In most Ethiopian TETC, students come from a rural area and live in town using a rent house. Through time and years of study, student’s awareness and knowledge about drugs and diseases might increase because of the Internet and social media access. The result showed that respondents aged 21–25 years were 2.47 times more likely to practice self-medication (AOR: 2.47, CI: 1.18–3.94, *p* < 0.05) than those aged 16–20 years. The current finding is in agreement with previous similar studies (Osemene and Lamikanra 2012; Gelayee 2017; Helal and Abou-ElWafa 2017). In contrast, some studies reported that no significant association between self-medication practice and the age of the participants was found (Bekele et al., 2016; Kassie et al., 2018).

In the present study, participants whose permanent residence was in urban areas tended to practice self-medication more often (AOR: 2.97, CI: 1.06–3.64, *p* < 0.05) than those who live in rural areas. This finding was in line with the study conducted in Egypt (Helal and Abou-ElWafa 2017). The possible explanation could be that those who lived in urban areas may have some awareness about the treatment and may have seen drug promotions, which may encourage them to practice self-medication rather than consulting the health professionals and visiting health institutions. Another reason for such a difference in practice related to residence might be related to the variance of accessibility to healthcare service. The logistic regression also showed that students whose parents had more than diploma certificate education level was 2.27 times more likely to practice self-medication (AOR: 2.27, CI: 1.32–3.71, *p* < 0.05) than those whose parents were unable to read and write. This finding is in agreement with a similar study conducted in Eretria (Araia et al., 2019). The possible explanation could be that those who had a diploma may have some awareness about drugs and diseases, and as a result, may encourage their children to practice self-medication rather than seeking healthcare institutions. However, a previous study was done in Serbia reported that the high level of parents’ education was independently associated with self-medication practice (Karthik et al., 2014).

Respondents who had access to the pharmacy were nearly two times more likely to practice self-medication (AOR: 2.12, CI: 1.43–4.46, *p* < 0.05) as compared to those who had not. This result is consistent with those of students in China (Wen et al., 2011), India (Divya et al., 2016), and Nigeria (Abdulraheem et al., 2016). This might be because most of the private pharmacies sold medication without a prescription and due to the lack of income and time to consult healthcare professionals. The current finding is supported by the inability to afford healthcare fees noted as the means for self-medication practice. The odds of self-medication practice among respondents who had peer/family pressure were 2.34 times more likely to practice self-medication (AOR: 2.34, CI: 1.53–3.56, *p* < 0.05) as compared to those who had not. The current result is consistent with those of studies carried out in China (Wen et al., 2011) and Uganda (Ocan et al., 2014). The possible explanation may be that friends/families were common sources of information about medication in developing countries like Ethiopia.

### Strengths and Limitations

This study had some limitations that should be taken into account when interpreting the results. As the study is cross-sectional and depends on self-reported assessment, underreporting is more likely to occur. This study did not include attitudes, awareness of the participants towards self-medication practice, social desirability, and types of medication. Even with the above limitations, the survey has a positive impact on the implications of health service in Ethiopia. Moreover, the study was multicentre and considered nonresponse participants. Finally, we recommended that future researches should consider participants’ attitudes, awareness, beliefs, and culture, which may be affected by self-medication practice.

## Conclusion

The result of the study revealed that two-thirds of the study participants practiced self-medication. Being from an urban area, having access to a private pharmacy, higher year of study, parents’ education level, and having peer/family pressure are significant factors for self-medication.

## Data Availability Statement

The raw data supporting the conclusions of this article will be made available by the authors, without undue reservation, to any qualified researcher.

## Ethics Statement

The studies involving human participants were reviewed and approved by the University of Gondar, Ethiopia. The patients/participants provided their written informed consent to participate in this study.

## Author Contributions

AM contributed to designing the study, writing the final research, data interpretation, data analysis, final manuscript preparation, and supervision of the study. MT and MG participated in data analysis and data interpretation. TA contributed to writing the final research, data interpretation, and manuscript preparation. All authors read and approved the final manuscript.

## Conflict of Interest

The authors declare that the research was conducted in the absence of any commercial or financial relationships that could be construed as a potential conflict of interest.

## Abbreviations

AOR, Adjusted odds ratio; COR, Crude odds ratio; ETB, Ethiopian birr; OTC, Over the counter; SPSS, Statistical package for social sciences
